# Prof. Parker’s Clinique

**Published:** 1847-04

**Authors:** 


11.—*Clinique of the College of Physicians and Surgeons.*—Prof. Parker.—Monday, February 1st, 1847.

*Case 2d.*—This man, 53 years old, has the aspect of one laboring under some constitutional difficulty; he has had a bad cough, which commenced about six years ago, in the fall. Now, Bronchitis usually comes on at this season, and remains till summer, but not unfrequently, continues in a chronic form for years. You will often meet with persons, who in the fall of every year will have an attack of this disease, and only recover when warm weather sets in. Such persons would be benefitted by going to a warm climate, where the temperature is agreeable; for it is not our excess of cold or warm weather that produces these difficuties, but it is the sudden change from one to the other that is so extremely prejudicial. The warm weather does not last long enough to restore the parts to a condition capable of resisting the influence of cold. He says he has no appetite, and that a fit of coughing often causes him to vomit: here we see the sympathy between the Lungs and the Stomach; the introduction of food into the Stomach irritates it; this irritation is transmitted to the Lungs by the Par Vagum, and coughing induced, which by mechanically irritating the Stomach, causes an evacuation of its contents; you will observe the same thing in Pertussis.

He complains of rheumatism: this generally attacks persons who have abused their digestive organs by excesses—especially intemperate people; cider, wine, and liquors, are supposed to have a specific influence in developing the complaint; but, as he has been a temperate man, we must refer it to some other cause. In my own opinion, rheumatism is not a local, but a constitutional affection; and in the treatment of it, I have obtained the happiest results, from simply attending to the digestive organs; bleeding has been highly extolled, but I think it valuable only as an adjuvant. The pain in this case is chiefly in the joints, which indicates it to be the Arthritic, and serves to distingush it from the Syphilitic form, which attacks the shafts of the bones.

On pressing his right side, he complains of pain, but neither percussion nor Auscultation indicates any trouble in his Lungs or Pleura; if there be any, it is rather on the left than the right; the pain, I suspect, is merely rheumatic, and in the Pectoral muscle. From all these symptoms, I am inclined to think it a case of Chronic Bronchitis, and would advise him to keep in his room, in which an even temperature should be maintained, and to attend to his digestive organs, by taking small doses of Cicuta and Blue Pill; and if his Stomach will bear it, the Balsam of Copaiva, which has a very beneficial influence on mucous membranes. If the irritation is very great, he would be benefitted by the Hydrocyanic Acid; these, with friction on the chest, will, I think be of much benefit to him.

*Case* 3*d.*—*Struma.*—A little girl, 10 years old, with an indurated gland on the neck, which was pronounced a true Scrofulous affection; it had inflamed, enlarged, softened, and finally become hard.

A poultice was ordered to be applied every night.

*Case* 4*th.*—*Struma.*—A man with Scrofulous disease in the bones of the little finger of the left hand; its removal was advised, consented to, and performed at the metacarpo-phalangeal articulation. The only difficulty to be encountered in this operation—the Professor remarked—was the lateral ligaments, which, to one who has not the anatomy of the parts in his mind, would prove very troublesome.

*Case* 5.—*Nævus Maternus*—situated just above the left eye—which has been treated twice with hot needles, and thereby much reduced; was quite soft at first, but now hard; this hardness will soon disappear. The Professor remarked, that from its situation, there was danger in the introduction of needles, of wounding the Supra-Orbital nerve, which if done, would be likely to produce Amaurosis.

